# Laundry wastewater treatment using a combination of sand filter, bio-char and teff straw media

**DOI:** 10.1038/s41598-019-54888-3

**Published:** 2019-12-10

**Authors:** Zaher Mundher Yaseen, Tibebu Tsegaye Zigale, Tiyasha, Ravi Kumar D., Sinan Q. Salih, Suyash Awasthi, Tran Minh Tung, Nadhir Al-Ansari, Suraj Kumar Bhagat

**Affiliations:** 1 Sustainable Developments in Civil Engineering Research Group, Faculty of Civil Engineering, Ton Duc Thang University, Ho Chi Minh City, Vietnam; 2 Department of Hydraulic and Water Resources Engineering, Institute of Technology, Ambo University, Ambo, Ethiopia; 3 Faculty of Civil Engineering, Ton Duc Thang University, Ho Chi Minh City, Vietnam; 4 Department of Civil Engineering, Institute of Technology, Ambo University, Ambo, Ethiopia; 5 Mechanical Engineering, Sam Higginbottom University of Agriculture, Technology And Sciences, Allahabad, India; 6 Institute of Research and Development, Duy Tan University, Da Nang, 550000 Vietnam; 7 Department of Civil Engineering, Gyan Ganga Institute of Technology and Sciences, Jabalpur, India; 8 Civil, Environmental and Natural Resources Engineering, Lulea University of Technology, Lulea, Sweden; 9 Research department, Agua International Water Relief, Los Angeles, California USA

**Keywords:** Environmental monitoring, Pollution remediation

## Abstract

Numerous researchers have expressed concern over the emerging water scarcity issues around the globe. Economic water scarcity is severe in the developing countries; thus, the use of inexpensive wastewater treatment strategies can help minimize this issue. An abundant amount of laundry wastewater (LWW) is generated daily and various wastewater treatment researches have been performed to achieve suitable techniques. This study addressed this issue by considering the economic perspective of the treatment technique through the selection of easily available materials. The proposed technique is a combination of locally available absorbent materials such as sand, biochar, and teff straw in a media. Biochar was prepared from eucalyptus wood, teff straw was derived from teff stem, and sand was obtained from indigenous crushed stones. In this study, the range of laundry wastewater flow rate was calculated as 6.23–17.58 m^3^/day; also studied were the efficiency of the media in terms of the removal percentage of contamination and the flux rate. The performances of biochar and teff straw were assessed based on the operation parameters and the percentage removal efficiency at different flux rates; the assessment showed 0.4 L/min flux rate to exhibit the maximum removal efficiency. Chemical oxygen demand, biological oxygen demand, and total alkalinity removal rate varied from 79% to ≥83%; total solids and total suspended solids showed 92% to ≥99% removal efficiency, while dissolved oxygen, total dissolved solids, pH, and electrical conductivity showed 22% to ≥62% removal efficiency. The optimum range of pH was evaluated between 5.8–7.1. The statistical analysis for finding the correlated matrix of laundry wastewater parameters showed the following correlations: COD (r = −0.84), TS (r = −0.83), and BOD (r = −0.81), while DO exhibited highest negative correlation. This study demonstrated the prospective of LWW treatment using inexpensive materials. The proposed treatment process involved low-cost materials and exhibited efficiency in the removal of contaminants; its operation is simple and can be reproduced in different scenarios.

## Introduction

Physical water scarcity and water stress have been a source of concern to the scientific community^1^. Water stress is more like an economic scarcity where there is a lack of economic support for the sufficient amount of water infrastructure which leads to the availability of inadequate amount of portable water. The decreasing level of uncontaminated water (water crisis) has pushed many scientists into developing techniques for reusing and recycling of contaminated water^2,3^. Numerous studies have been conducted on the present and future water scarcity situation^4,5^. A couple of studies reported that Ethiopia, Eritrea, Nigeria, Uganda, Tanzania, Niger, and Somalia will have renewable water resources below the calculated threshold of 1500 m^3^/capita/year by the year 2030^6,7^. Ethiopia comes under the economically scarce zone along with most of the African countries where water poverty index (WPI 35–48) was reported severe including surface water index (SWI = −0.95 to −0.90) and groundwater index (GWI = −0.75 to −0.50)^6,7^; this desperate situation drives the need for some important measures to be developed and among such measures, wastewater treatment and its reusage remains the promising solution.

Wastewater treatment is an ancient technique but the necessity of saving water and wastewater treatment have been felt in the last few decades due to the increasing water pollution and water demand^8,9^. Wastewater has been divided according to the source of generation; therefore, laundry wastewater (LWW) is one of the sources among others^8^. Studies have reported the average daily LWW effluents discharge from textile industries, high rise apartments, low rise apartments, bars, hotels & hospitals as 189 L, 246 L, 1280 L, 2080 L, and 624 L, respectively^10,11^.

Laundry wastewater is generated during the washing of clothes at household and industrial levels; the pH of household and industrial LLW is approximately 5.6, with chemical oxygen demands (COD) of almost 4800 mg∙L^−1 ^^12^, while LLW of hotels has COD value ranging from 400–1200 mg∙L^−1 ^^13^. Laundry wastewater also contains average total suspended solids of 0.08 mg∙L^−1^, iron (0.037 to 0.72 mg∙L^−1^), and phosphate (94.65 mg∙L^−1^). Linear alkylbenzene sulfonates (LAS) was detected (12.24 to 1023.7 mg∙L^−1^) in 30 different LLW samples from Brazilian commercial facilities^12^. LAS has been found in downstream from raw sewage outfalls in different receiving bodies, such as river^14^. Some of these pollutants had been found threatening the local environment of disposal site, as well as toxic to the human^15^. Furthermore, LAS has a promising characteristic, i.e., inhibition of biological activities^16^.

The study aims to address the issue of economic water scarcity by proposing an economic method for LWW treatment. Additionally, the treatment of the significant amount of LWW generated per day in the study area will minimizes the contamination of the disposal sites and make them useful for other purposes. Various contemporary techniques have been discovered, nonetheless, very few are applicable in developing countries^8,12,17^. Sedimentation and filtration have been used to treat LWW but it only reduces the pH, total suspended solids (TSS), total dissolved solids (TDS), turbidity and total hardness^17^. There are no changes in COD and biochemical oxygen demand (BOD), while iron content has been reported to increase by 93%. However, the technique is economical and could be reused for first rinse of dirty clothes only^17^. Slanted soil treatment system with plastic foam tray and kanuma soil which have alumina and hydrated silica has shown good performance as the observed removal percentages of suspended solids (SS), BOD, COD, total nitrogen and total phosphorus were 78%, 83%, 85%, 78%, and 86%, respectively^18^. Biochar produced from natural adsorbents is cheaper than activated carbon if produced from waste wood, bagasse fly ash, coconut-hay carb, and peat^19^.

Moreover, the utilization of easy and locally available material will encourage the application of this research in other developing countries. In line with this, bio-char sourced from local woods was considered a good option to ensure cost-effectiveness. Biochar is the potential absorbent for absorbing synthetic organic carbon from wastewater; it was produced under controlled pyrolysis temperature, i.e. 700 °C^20,21^. The morphological study of bio-char showed a promising surface of >500 m^2^/g; this large surface area will be great for the adsorption of wastewater contaminants.

Extensive studies have been performed on the indigenous and freely accessible adsorbent (i.e., teff straw which is one of the media in the study) since it is a part of the staple food of Ethiopian households. The treated straw can be used for the treatment of domestic and industrial wastewater at the lowest cost^22–24^. Desta Mulu Berhe, (2013) examined treated and untreated teff straw adsorbents for the textile effluent treatment and reported them as potential adsorbents for Cr, Ni, and Cu removal^23^. On the other side, Tadesse *et al*., (2015) demonstrated the effectiveness of agriculture waste materials as adsorbents; the study used teff adsorbent together with wheat bran, almond shell, coconut tree sawdust, and rice straw for the quantitative removal of Cr(VI) from leather industry wastewater. The authors presented the Langmuir isotherm (R^2^ = 0.9739) and pseudo-second order model (R^2^ = 0.9999) which revealed the approach as a suitable wastewater treatment method^22^. Wassie and Srivastava, (2016) reported teff straw as a mesoporous material with a pore diameter of 43.9 Å and surface area of 30.5 m^2^/g using scanning electron microscopy (SEM) analysis. They used this material for Cr(VI) metal removal from aqueous solution^24^.

In this study, a multi-barrier technique with a new multi-media combination was tested for laundry wastewater treatment. The multi barrier holistic approach proposed in this study uses three media barriers consisting of sand, bio-char, and teff-straw. These three steps of treatment will reduce the chances of treatment failure, increase the amount of contamination removal at each stage, and increase the overall efficiency of the treatment process. To the best of the authors’ knowledge, the suggested combination of specific locally available media has not been explored in any previous studies in the field of LWW treatment.

This study is presented in sections; the second section described the materials and methods used in this study while the third section discussed the results of the investigations. The last section presented the conclusions drawn from the results of the study.

## Materials and Methods

### Study area description

Various regions of Ethiopia are characterized with severe water shortage, coupled with sediments and pesticide ingress into the aquifers. This has made life difficult for the rural people in such regions^25,26^. Accessible water is traced with high amounts of TS, total nitrogen (TN), total phosphorus (TP), as well as synthetic surfactants as they are used in high amount in developing countries such as Ethiopia^27,28^. It is worth to mention that Ethiopia is a low income country as per the world economic forum; the World Bank classified Ethiopia as one of the poorest countries, with a yearly per capita income of $783^29,30^. In the case of higher education and research, Ethiopia faces several challenges over time and up to date^31–33^. Consequently, there is a lack of laboratory facilities to estimate the surfactant or LAS contamination levels owing to the cost implication, lack of budget for sending samples to external laboratories, and the lack of expertise in this field^34–36^.

This study was conducted at the Institute of Technology (IOT), Ambo University, Ethiopia. Ambo is located in the West Shewa zone of the Oromia region, 125 km west of Addis Ababa. The IOT is one of the campuses of the Ambo University which is dedicated to 13 engineering departments, housing 6186 students in 2018. There are 36 dormitories in this campus, and each has its own laundry area. The LWW was collected from the washing platform of the female dormitories which accommodates 1363 female students of the campus. The LWW collected from this area was sampled, characterized and treated. To estimate the per capita demand for laundry purpose, the total demand for water supply was obtained from the office of Ambo Urban Water Supply and Sewerage Service Enterprise (AUWSSSE). The total population of the IOT campus was obtained from the Registrar and Human Resource office of the campus.

### Flow rate measurement

LWW was collected during hand washing of female clothes and shoes by different kinds of anionic detergent powder. Normally, LWW is collected in sewer and disposed to the nearest public river i.e., Alaltu. To establish the pattern of LWW discharge during a week, flow rates were manually measured using jar and cylinder with a stopwatch. Each experiment was done twice, and the average value was documented. The experiment  was recalculated whenever the experimental error exceedsed ±5%. The flow rate was calculated in m^3^/day by applying in Eq. 1. The liter per capita demand for laundry was calculated using Eq. 2.

1 $$\frac{LWW\,in\,liter}{hour}\times \frac{total\,washing\,hour}{day}=\frac{LWW\,in\,liter}{day}\times \frac{1}{1000}=\frac{{m}^{3}}{day}$$

2 $$\frac{{\rm{an}}\,{\rm{average}}\,{\rm{flow}}\,{\rm{rate}}\,{\rm{of}}\,{\rm{a}}\,{\rm{month}}}{{\rm{Total}}\,{\rm{number}}\,{\rm{of}}\,{\rm{users}}}={\rm{Liter}}\,{\rm{per}}\,{\rm{capita}}\,{\rm{demand}}\,{\rm{for}}\,{\rm{laundry}}$$

### Analytical method

The sample (100 L of LWW each time) was collected in clean and sanitized polyethylene sample bottles at different times and days of a week from different washing cycles, mixed and stored in a refrigerator at 4 °C. Prior to store, the feed was filtered through a 5 μm polypropylene depth filter to remove coarse solids and fibrous materials. The feed was analyzed before filtration and after filtration and the outcome of the analysis was presented in the results section. Temperature and pH were measured on-site using a portable instrument (HI98128). The other parameters of the sample (TDS, TSS, TS, electrical conductivity (EC), Total Alkalinity (TA), DO, BOD and COD) were estimated using the relevant standard methods (APHA 1999 – by The American Public Health Association, American Water Works Association, Water Environment Federation). Each experiment was executed thrice, and the average value was reported. In case of experimental error greater than ± 5%, the experiment was repeated. The percentage removal of COD, BOD, DO, TS, EC, TA, and pH from the solution was calculated using Eq. 3^24^.

3 $$Removal\,efficiency\,( \% )=\frac{{C}_{0}-{C}_{t}}{{C}_{0}}100$$

where, C_0_ and C_t_ represents the initial and final individual parameter concentration in mg∙L^−1^ except EC which is in µS/cm, and ‘t’ represents the biosorption time.

### Correlation Matrix and the statistical indicators

The WQ data was normalized using scaling before correlation analysis for the best statistical calculation. The data was normalized using Eq. 4.

4 $${\rm{Normalized}}\,{\rm{data}}=log(x)$$

where *x* is the water quality parameters. The Pearson correlation coefficient (*r*) was calculated to establish the influence of the other WQ variables on DO using Eq. 5.

5 $$r(a,b)=E(ab)/(\sigma a)\,(\sigma b)$$

where, *E*(*ab*) is the cross-correlation between a and b, and *σa and σb* are the variances of the signals a and b^37^. The statistical analysis was done using RStudio (package “psych” version 3.6.1).

### Media Preparation

To construct the filter, different layers of sand and gravel were arranged systematically. Different sizes of gravel, coarse and fine sand were collected from crushed stone process. Crushed stone is preferable than river sand to minimize the biological contamination risk. To achieve the maximum efficiency of media, it is important to prepare the filter media by following all steps. To eliminate any dirt in the filter media, it was washed thoroughly before sieving. The sand and gravels were sorted into the required size as listed in Table 1^20,21,23,24,38^. The selection of filter media was based on locally available materials. The size of gravel for the construction of base material was chosen to be 5–10 mm.

**Table 1:** Characteristics of each layer of media used in a filter.

Media	Size in mm	Dimension in mm^a^	Source	^b^BET Surface area in m^2^/g
*Gravel for base material*	5–10	250 × 250 × 250	Crushed stone	NA
*Coarse sand for separation*	2.5–5	250 × 250 × 250	Crushed Stone	NA
*Fine sand*	0.1–2.5	250 × 250 × 550	Crushed Stone	NA
*Crushed Bio-char*	1–5^c^	250 × 250 × 550	*E. camaldulensis* and *E. globulus*	400–600
*Teff-straw*	0.25–1.0^d^	250 × 250 × 550	*Eragrostis tef*	30.5

length X width X height; ^b^Brunauer–Emmett–Teller (BET), ^c^(Joshua P Kearns *et al*., 2014; Joshua P. Kearns *et al*., 2014; Kearns *et al*., 2015), ^d^(Desta Mulu Berhe and Desta, 2013; Wassie and Srivastava, 2016).

**Figure 1:**
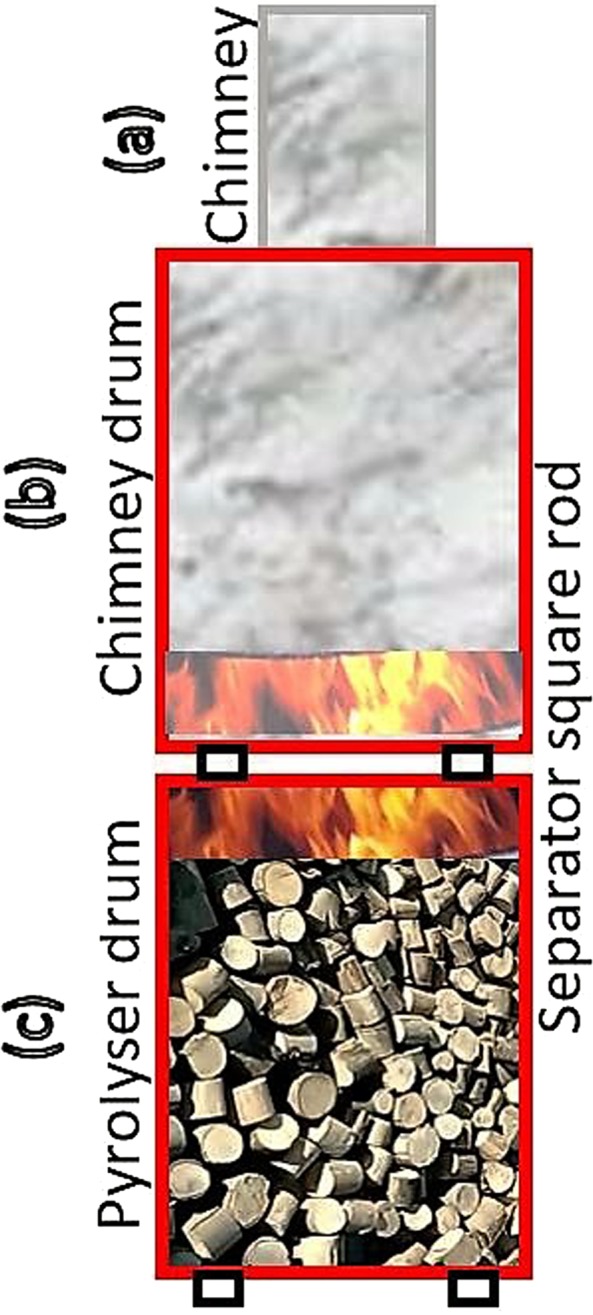
Setup for producing the bio-char: Two drum (**b,c**) where used which were filled with chopped eucalyptus wood chips (5–10 cm thickness, 8–13 cm length) and burnt till the temperature reaches between 550–850 °C for 2 hours in pyrolyzer drum. (**a**) When chimney starts producing white smoke is the indicator for the biochar production completion.

The preparation of biochar was done under a technology transfer workshop conducted by Agua International Water Relief, United States (http://www.aguaint.org) at IOT, Ambo University. Locally available dry woods, such as *E. camaldulensis* and *E. globulus* (commonly known as Eucalyptus, 5–10 cm thickness, 8–13 cm length) were used to produce the biochar as shown in Fig. 1. The detailed process of biochar production can be downloaded from Aqueous solutions (http://www.aqsolutions.org). Within 2 hours of burning, the temperature of the furnace reached 550–850 °C in the pyrolyzer drum (Fig. 1) which helped to achieve the required surface area and pore size for filtration^20^. During this period, the color of the flame changed from bright orange to faint blue (visible between the drums). Visible smoke from the chimney signals the completion of the process. Water was continuously sprayed on the drum to reduce the exterior heat. The char of the pyrolyzed drum was taken out; wet quench generated the steam which again helped to open the pores of the biochar in a substantial way and increased its adsorption capacity^21,39^. The char was crushed and sieved to a uniform size of 1–5 mm which has a greater exposed surface area and shorter travel distance for contaminants diffusing into the char pores^20^.

Among the three types of teff straw, i.e. white, brown and red, brown teff straw (*Eragrostis tef*) was used for this study. Teff straw is a low-cost biomaterial locally available in Ethiopia^22,23^. Teff straw served as a potential absorber for different contaminations, such as Cr(VI) and phenol red from wastewater^24,40^. The teff straw was decanted, washed with double distilled water to remove all dirt, and sieved to 0.25–1.0 mm size for efficient adsorption^23,24^.

### Experimental setup

**Figure 2:**
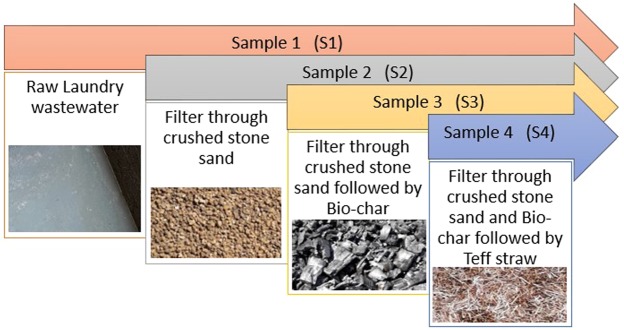
A flow diagram of experimental setup of the filter: The raw water in S1 was allowed to collect in upward direction to discharge into S2 chamber where gravitational flow took place though sand media and then discharge into S3 in the same way as previous then gravitation flow reaches to upward discharge pipe to transfer the treated water to S4 chamber where led to gravitation flow through teff straw and effluent occurred in the upward direction.

This study was performed in a laboratory-scale set-up to test the efficiency of the filter in treating LLW. Four gallons (100 L) were cleaned; the first gallon was set as the storage and the other three gallons were fitted with bottom drainage gravel. This was followed by the separation gravel, and then, up-flow gravel roughing filter, followed by a down-flow biologically-active sand (second gallon), bio-char (third gallon), and teff straw (fourth gallon) filter for filtration processes (Fig. 2). The samples were taken and tested after each step of the filter setup. Four different samples were taken each time to make a comparative analysis of the treated LWW from different filter media. Figure 2 showed the samples taken from different filter systems (S1 = raw laundry wastewater, S2 = filtered through the locally-available crushed stone sand, S3 = filtered through biochar, and S4 = filtered through teff straw).

## Results and Discussion

### Laundry wastewater discharge

The results of this study elucidated two important observations, including the maximum weekly flow rate and the average laundry water demand. Table 2 illustrated the average flow rate from Sunday to Saturday. The total average flow rates were calculated for the next seven days, followed by the quantitative and qualitative analyses.

**Table 2:** A total average flow rate of the LWW discharge (The average discharge of each day of a week was collected and total average discharge is for the same day of a month).

Days	Average discharge each day (m^3^/day)				Total Average Discharge on a specific day (m^3^/day)
*Sunday*	5.84	6.55	6.35	6.17	6.23
*Monday*	8.96	8.37	7.63	8.04	8.25
*Tuesday*	10.49	10.59	10.05	10.80	10.48
*Wednesday*	7.58	6.97	7.20	7.71	7.37
*Thursday*	8.47	8.15	7.71	7.58	7.98
*Friday*	8.47	10.85	9.00	7.58	8.98
*Saturday*	16.36	17.85	18.38	17.70	17.58

**Figure 3:**
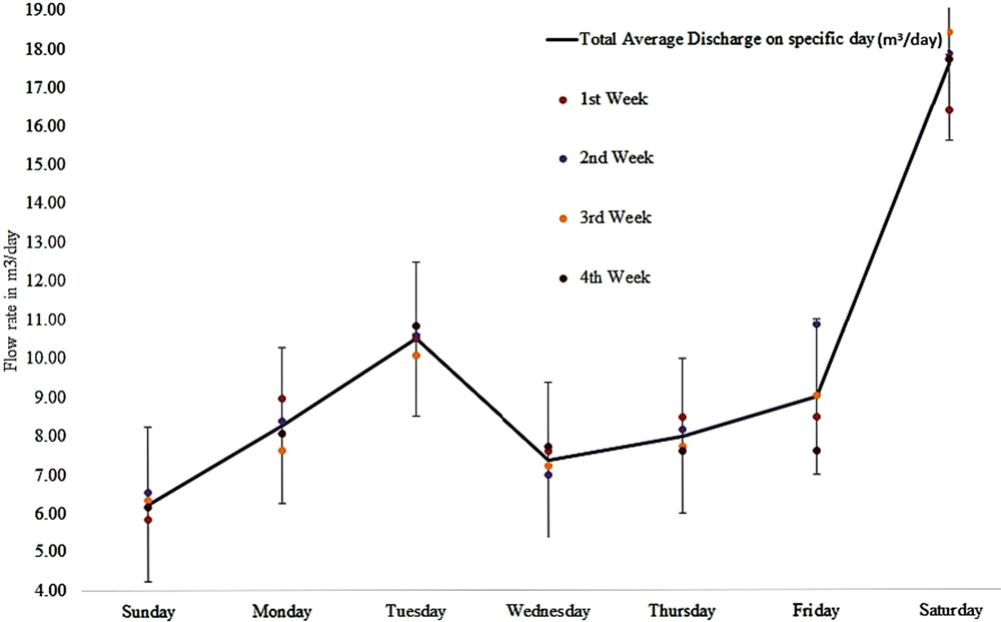
Time series peak flow rate at daily basis measured and analyzed total average and peak discharge.

Figure 3 displays the substantial average flow rate of each day of a week. The maximum flow rate was observed on Saturdays and this was because students consume more water on this day for their laundry purposes. Quantitatively, the minimum flow rate was observed on Sunday (6.23 m^3^/day) while the maximum flow rate was observed on Saturday (17.58 m^3^/day). The second maximum flow rate was observed on Tuesday due to the gap of a weekend in which most students are exposed to dirt owing to their busy activities at the local markets buying their stuffs for the whole week. They normally attend classes on the first day of the week and may decide to take rest for laundry on the following day. Figure 3 showed the total average flow rate for each day of the week with 5% error bar. The average LWW produced per month estimated by using Eq. 2 was approximately 1476.66 m^3^.

The liter per capita demand for laundry was determined as 0.007 m^3^/day or 7 L/day. The total water demand of the campus was 28000 m^3^/month (considering the data taken from the Human Resource office of the university), comprising of 18.6% leakage, 30% construction work, and 10% others. This amounts to the total per capita demand of 0.055 m^3^/day or 55 L/day which is low compared to 42–45 Liters/unit/day reported for the Institute of Hong Kong and Jordan^41,42^. There is about 2.7 times higher water demand per capita than the World Health Organization (WHO) standard for urban areas of Ethiopia^43^. Hence, 5.3% laundry water demand was estimated from the total water demand of the university. However, 18.6% water loss of the total water intake per month in the campus have been reported in a thesis submitted by a graduating research student^44,45^.

### Removal efficiency

The results of the pilot prototype were promising when considering the price, availability of the raw materials, and the quality of the water after treatment. Intensive analytical assessments were performed on the treated LWW samples from each treatment step as listed in Table 3.

**Table 3:** A characterization of laundry wastewater for S1, S2, S3 and S4 in terms of TS (mg∙L^−1^), TSS (mg∙L^−1^), TDS (mg∙L^−1^), pH, EC (µS/cm), Total Alkalinity mg∙L^−1^ (CaCO_3_), DO (mg∙L^−1^), COD (mg∙L^−1^), BOD^5^ (mg∙L^−1^).

	TS (mg∙L^−1^)	TSS (mg∙L^−1^)	TDS (mg.L^-1^)	pH	EC (µS.cm^-1^)	Total Alkalinity mg∙L^−1^ (CaCO_3_)	DO (mg∙L^−1^)	COD (mg∙L^−1^)	BOD^5^(mg∙L^−1^)
S1	3194.4	2666.4	528	8.32	648.8	579.6	2.67	4832	2222.72
S2	327.2	136	191.2	6.94	299.2	196.56	3.91	1056	485.76
S3	370.4	186.4	184	7.02	315.2	168	4.27	1360	625.6
S4	226.4	26.4	200	6.4	347.2	120	5.4	832	382.72
S1	2395.8	2011.6	384.2	7.8	540.4	720.4	3.8	3624	1674
S2	301.5	145.2	156.3	7.3	280.1	240.5	4.2	792	364
S3	350.4	178.6	171.8	7.4	284.2	210	4.9	1020	370
S4	202.8	20.1	182.7	6.1	425.5	150.5	5.8	624	495
S1	3050.6	2404.2	646.4	8	570.6	700.4	3.5	4010	1954
S2	375.2	135.6	239.6	7.1	285.5	215.1	4.1	953	425
S3	404.2	192.3	211.9	7	325.1	195.6	4.5	1250	405
S4	220	23.5	196.5	5.8	401.8	135.2	5.4	795	478
S1	3450.2	2301.1	1149.1	8.4	548.4	490.5	3.1	4950	2245
S2	320.5	110.4	210.1	6.9	354.5	205.5	3.6	1024	505
S3	385.5	210.2	175.3	6.9	365.5	135.5	4.1	1395	492
S4	245.4	27.7	217.7	6.4	385.1	115.5	5.9	820	310
S1	3594	2841.2	752.8	8.5	740.4	495.6	1.8	6040	2770
S2	330.4	154.8	175.6	6.9	342.5	200.1	3.8	1265	486
S3	390.8	210.5	180.3	7.1	340.5	165.1	4.2	1300	445
S4	265.4	29.4	236	6.7	360.1	101.5	6.1	1040	287
S1	3954.4	2945.5	1008.9	8.8	701.5	567.2	2.2	5150	2512
S2	335.1	175.2	159.9	7.1	310.1	170.2	4	1295	440
S3	400.4	140.8	259.6	6.9	295.5	142.5	4.9	1125	365
S4	185.5	24.5	161	6.9	345.8	101.1	5.8	940	495
S1	3656.5	2793.4	863.1	8.5	645.5	550.2	2.5	4010	2135
S2	323.4	140.5	182.9	7.4	300.4	183.4	3.9	1320	475
S3	350.1	158.7	191.4	7.1	308.7	165.5	4.5	1485	425
S4	224.6	26.5	198.1	7.1	341.7	108.5	5.9	835	345

**Figure 4:**
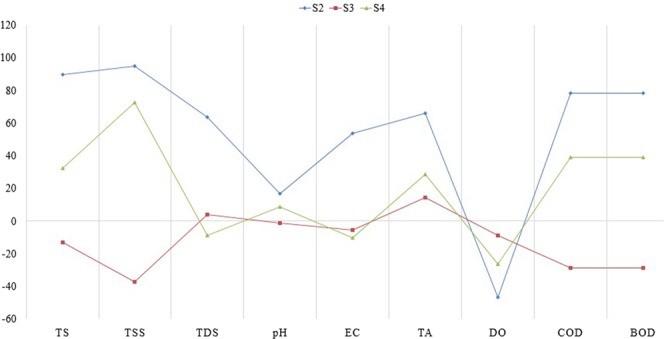
Differences in removal efficiency of S2, S3 and S4 filtration for each LWW parameters.

Figure 4 illustrated the removal efficiency of all the tested parameters of LWW. S1 served as the feed to S2, and so on. S3 and S4 presented high removal percentages due to their significant adsorbent behavior as earlier reported in the previous studies^21,22^. Among several types of wastewater filter, sand filter plays a major role in the removal of solids and improvement of pH level^46–48^. Biochar and teff straw showed significant adsorbing capacities by presenting the maximum LWW contaminants removal efficiency. Regarding EC and COD, the decline in their removal could be due to the presence of highly concentrated synthetic compounds such as alcohol ethoxylate (AEO) and LAS in the solution^12,49^.

### Effect of filtration flux

**Figure 5:**
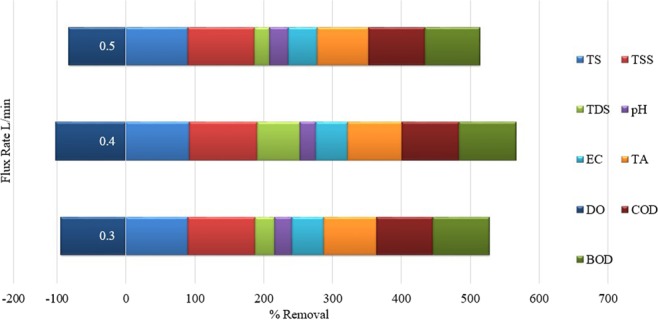
Percentage removal vs three different flux rate for all LWW measured characteristics.

The effects of flux rate at 0.3, 0.4, and 0.5 L/min on the percentage removal of each parameter of LWW was determined (Fig. 5). During the testing of the filters, the flux rate was varied to evaluate the best possible flow rate that will give the maximum percentage removal of the contaminants. It was observed that a flux rate of 0.4 L/min had the highest percentage removal, whereas 0.3 L/min and 0.5 L/min were less effective. Low flux rate promoted cake layer formation, thereby slowing the filtration process. On the other hand, 0.5 L/min flux rate enhanced the turbulence level of the sample and reduced the cake layer formation on the top layer of media. Among the three tested flux rates, 0.4 L/min showed the best pollutant removal performance from the sample as it maintained a substantial retention period to promote the removal efficiency^50^.

### Correlation of various LWW parameters

**Figure 6:**
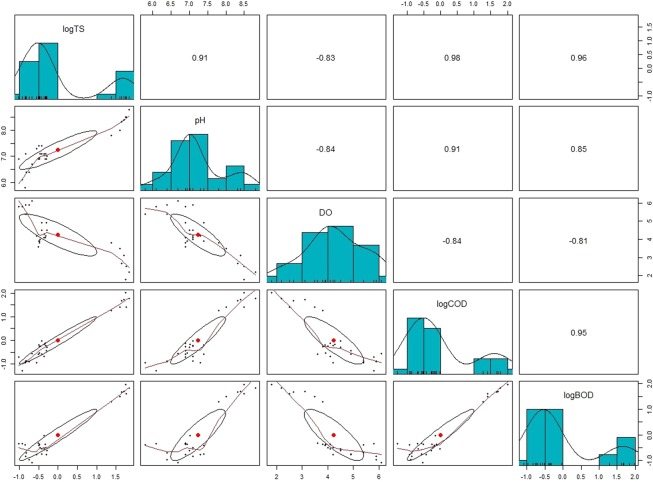
Multi-variate mixed correlation plot: The scattered plots have correlation elipse which presents the maximum concentration of the value along with implementation of robust fitting with lowess to visualize the relationship between the variables and changing trends.

The results of the LWW quality tested before and after water treatment displayed using multi-level plots as reported in Fig. 6. The plot presents the scatter plot of matrices along with bivariate scatter plot below diagonal, histograms on the diagonal and Pearson correlation above the diagonal. To understand the relation between TS, pH, DO, COD and BOD each parameter was described separately. The scattered plots exhibited correlation elipse that presents the maximum concentration of the value along with implementation of robust fitting with lowes to visualize the relationship between the variables and changing trends.

Being that the direction of the reaction and the active function of the sorbents are directed by the pH, the absorption and change in water quality are highly affected by the pH. In this study, the pH was found to relate inversely with DO (r = −0.84); the highest amount of DO (6.1) was found at pH 6.7. COD, BOD, and TS removal were maximum between pH 7–7.4, 6.4–7.1, and 5.8–7.1, respectively; thus, the best LWW treatment performance was achieved between pH 5.8–7.1.

DO greatly affects the TS, COD, and BOD concentration. DO was found to be inversely proportional to TS (r = −0.83), COD (r = −0.84), and BOD (r = −0.81) which implies that an increase in DO will simultaneously decrease the whole parameters. Mini-max analysis revealed that the range of DO of untreated LWW (S1) and treated LWW (S4) was (1.8–3.8) and (5.4–6.1) mg∙L^−1^, respectively; similarly, the showed that, with respect to S1 and S4, the values of TS, COD, and BOD ranged from (2395.8–3954.4), (4010–6040), and (2223–2770) mg∙L^−1^ to (185.5–265.4), (624–1040), and (287–495) mg∙L^−1^, respectively. The histogram clearly showed an increase in the COD, BOD and TS concentration at the last stage of the treatment, making it clear the teff straw and impurities affected the quality of the water.

### Reclamation of treated water

Treated LWW can be disposed to water bodies or reutilized. As per Table 4, it can be seen that the other techniques were efficient in surfactant or LAS removal and in controlling the levels of the pH, TS, TSS, TDS, EC, TA, and COD^2,13,17,51–56^. However, these techniques are either expensive or time-consuming and their efficiency declines with time. Table 4 also showed some of the cost-efficient techniques developed over the last couple of years. The proposed approach reduces the levels of TS, TSS, TDS, pH, EC, TA, COD, and BOD in a manner that will support the significant removal of surfactant or LAS.

**Table 4:** Result of different LWWT studies by using various treatment techniques and their limitations.

	TS (mg∙L^−1^)	TSS (mg∙L^−1^)	TDS (mg.L^-1^)	pH	EC (µS.cm^-1^)	Total Alkalinity mg∙L^−1^ (CaCO_3_)	DO (mg∙L^−1^)	COD (mg∙L^−1^)	BOD^5^ (mg∙L^−1^)	Surfactants/LAS	Reference
Combined coagulation/flocculation/sedimentation process (C/F/S) and membrane separation	500	—	435	6.8	278	—	—	83	—	5.1/NA	(Nascimento *et al*., 2019)
Electrocoagulation/Electroflotation	—	3	—	5.9	1.4 (mS/cm)	11	—	80	—	Methylene Blue Active Substances – MBAS = 5.3	(Dimoglo *et al*., 2019)
Modified laundry waste water treatment system	—	40	380	7.7	—	—	—	310	40	—	(Ahmad and EL-Dessouky, 2008)
Granular Activated Carbon (GAC)	—	4	—	7.4	1275	—	—	140	—	1.6/NA	(Ciabattia *et al*., 2009)
Hybrid System (MF and UF) with 157 days total operation time	—	1.55	—	—	—	—	—	145–260	—	0.04/NA	(Babaei *et al*., 2019)
Expanded granular sludge bed (EGSB) with 84 days of operational time	5.79	—	—	8.0 ± 0.2	—	331 ± 99	—	68 ± 17	—	NA/ 3.2 ± 1.7	(Faria *et al*., 2019)
A combined process of the up-flow multi-medium biological aerated filter (UMBAF) and the multi-media biological aerated filter (MBAF); 25% declination of LAS removal within 24 days.	—	—	—		—	—	—	35	—	7	(Ji *et al*., 2019)
Vertical-Subsurface Flow Constructed Wetland system	—	22.1–32.8	722–783	7.8	—	—	—	926–113	182–43	—	(Watiniasih *et al*., 2019)
Moringa oleifera Seeds	5.77–7.01	0.37–0.11	—	—	—	—	—	277–313	—	—	(Al-Gheethi *et al*., 2017)
Integrated of sand-biochar-teffstraw	224.3	25.4	198.9	6.5	372.5	118.9	5.8	840.8	398.9	—	This study

At the flux rate of 0.4 L/min, the removal efficiency was very effective. The qualitative analysis of S4 showed a significant change in the quality of the wastewater. The levels of TS, TSS, and TDS of S4 were under the standard permissible limits for irrigation^57,58^. For the survival of aquatic life, DO is required at a minimum level of 4 mg∙L^−1^ or above at 20°C and this is the average temperature in Ethiopia^59,60^. The DO of S1 was also found to be 2.8 mg∙L^−1^, making it unsuitable to be disposed into the river. Therefore, LWW needs treatment so that it can be directly disposed into water bodies. The observed maximum removal rates of TS and TSS were 92% to ≥99%, while COD, BOD, and alkalinity removal rates were found in the range of 79% to ≥83%. The minimum removal rates were those of DO, TDS, pH, and EC (22% to ≥62%).

The achieved wastewater treatment results demonstrated an optimistic  use of treated LWW for different purposes. Previously, treated grey water from confined trench has been reused for the irrigation of olive trees and some vegetable crops^61,62^. In recent years, short-chain fatty acids and methane production can be achieved reusing LWW which contains a controlled amount of LAS^63,64^. Accordingly, it is recommended to have a prior pilot research to check the surfactant or LAS level before preferring to reutilize LWW for the mentioned purposes.

## Conclusion

This paper investigated the feasibility of treating LWW with cost-effective and locally-available materials. The results of this study can be applicable to other developing countries where there is a need to conserve a significant amount of water and protect water bodies from contamination. This study fabricated an integrated LWW treatment system and evaluated its performance efficiency. The treatment technique uses a sequence of up-flow gravel roughing filter, followed by a down-flow biologically-active sand, bio-char, and teff straw which have a great filtration capacity. The results of this study showed the maximum flow rate of 17.58 m^3^/day and the minimum flow rate of 6.23 m^3^/day to be found on Saturday and Sunday, respectively. The average water demand for laundry was found to be 7 L/day, which implies the generation of around 12059 m^3^ of wastewater per year (during college working days). This huge amount of LWW is usually discharged directly into the sewer and water bodies, causing harm to the environment. The flux rate of 0.4 L/min performed the best in controlling the levels of TS, pH, DO, COD, and BOD. This result demonstrates that the selected media can efficiently remove pollutants from LWW with a high level of removal performance. This study serves as a guide towards wastewater reclamation in areas where low water quality is permissible for other activities, such as for flushing, gardening, and construction purposes to save poratable water^13,61^. Reusing wastewater can save a huge amount of good quality water and extend the life of other water sources.

## References

[1] Hasan E, Tarhule A, Hong Y, Moore B (2019). Assessment of physical water scarcity in Africa using GRACE and TRMM satellite data. Remote Sens..

[2] Dimoglo A, Sevim-Elibol P, Dinç Ö, Gökmen K, Erdoğan H (2019). Electrocoagulation/electroflotation as a combined process for the laundry wastewater purification and reuse. J. Water Process Eng..

[3] Yu ZLT, Rahardianto A, DeShazo JR, Stenstrom MK, Cohen Y (2013). Critical review: regulatory incentives and impediments for onsite graywater reuse in the United States. Water Environ. Res..

[4] Falkenmark, M. *et al*. *On the verge of a new water scarcity: a call for good governance and human ingenuity*. (Stockholm International Water Institute (SIWI), 2007).

[5] Mungkung, R., Gheewala, S. H., Silalertruksa, T. & Dangsiri, S. Water footprint inventory database of Thai rice farming for water policy decisions and water scarcity footprint label. *Int. J. Life Cycle Assess*. 1–12 (2019).

[6] Yang, H., Reichert, P., Abbaspour, K. C. & Zehnder, A. J. B. A water resources threshold and its implications for food security. (2003). 10.1021/es0263689 12901649

[7] Raghavendra S, Deka PC (2014). Support vector machine applications in the field of hydrology: A review. Appl. Soft Comput. J..

[8] Tchobanoglous G, Burton FL, Stensel HD (1991). Wastewater engineering. Management.

[9] Henze, M., van Loosdrecht, M. C. M., Ekama, G. A. & Brdjanovic, D. *Biological wastewater treatment*. (IWA publishing, 2008).

[10] Bhagat SK, Tiyasha, Bekele DN (2018). Economical Approaches for the Treatment and Reutilization of Laundry Wastewater - a Review. J. Ind. Pollut. Control.

[11] Bhagat SK, Tiyasha (2013). Impact of millions of tones of effluent of textile industries: analysis of textile industries effluents in Bhilwara and an approach with bioremediation. Int. J. ChemTech Res..

[12] Braga JK, Varesche MBA (2014). Commercial Laundry Water Characterisation. Am. J. Anal. Chem..

[13] Ciabattia I, Cesaro F, Faralli L, Fatarella E, Tognotti F (2009). Demonstration of a treatment system for purification and reuse of laundry wastewater. Desalination.

[14] Petrovic M, Barceló D (2003). Occurrence of surfactants in the environment. Compr. Anal. Chem..

[15] Kern DI (2013). Toxicity and genotoxicity of hospital laundry wastewaters treated with photocatalytic ozonation. Sci. Total Environ..

[16] Elsgaard L, Petersen SO, Debosz K (2001). Effects and risk assessment of linear alkylbenzene sulfonates in agricultural soil. 1. Short-term effects on soil microbiology. Environ. Toxicol. Chem. An Int. J..

[17] Ahmad J, EL-Dessouky H (2008). Design of a modified low cost treatment system for the recycling and reuse of laundry waste water. Resour. Conserv. Recycl..

[18] Itayama T (2006). On site experiments of the slanted soil treatment systems for domestic gray water. Water Sci. Technol..

[19] Eremina AO, Golovina VV, Ugai MY, Rudkovskii AV (2004). Activated carbons from waste wood in wastewater treatment to remove surfactants. Russ. J. Appl. Chem..

[20] Kearns JP, Knappe DRU, Summers RS (2014). Synthetic organic water contaminants in developing communities: an overlooked challenge addressed by adsorption with locally generated char. J. Water, Sanit. Hyg. Dev..

[21] Kearns JP, Knappe DRU, Summers RS (2015). Feasibility of Using Traditional Kiln Charcoals in Low-Cost Water Treatment: Role of Pyrolysis Conditions on 2,4-D Herbicide Adsorption. Environ. Eng. Sci..

[22] Tadesse B, Teju E, Megersa N (2015). The Teff straw: a novel low-cost adsorbent for quantitative removal of Cr (VI) from contaminated aqueous samples. Desalin. Water Treat..

[23] Desta Mulu B, Desta MB (2013). Batch Sorption Experiments: Langmuir and Freundlich Isotherm Studies for the Adsorption of Textile Metal Ions onto Teff Straw (Eragrostis tef) Agricultural Waste. J. Thermodyn..

[24] Wassie AB, Srivastava VC (2016). Teff straw characterization and utilization for chromium removal from wastewater: Kinetics, isotherm and thermodynamic modelling. J. Environ. Chem. Eng..

[25] Teklu BM, Adriaanse PI, Ter Horst MMS, Deneer JW, Van den Brink PJ (2015). Surface water risk assessment of pesticides in Ethiopia. Sci. Total Environ..

[26] Abiye TA, Sulieman H, Ayalew M (2009). Use of treated wastewater for managed aquifer recharge in highly populated urban centers: a case study in Addis Ababa, Ethiopia. Environ. Geol..

[27] Angassa K, Leta S, Mulat W, Kloos H, Meers E (2019). Effect of hydraulic loading on bioremediation of municipal wastewater using constructed wetland planted with vetiver grass, Addis Ababa, Ethiopia. Nanotechnol. Environ. Eng..

[28] Ayele L, Pérez-Pariente J, Chebude Y, Diaz I (2016). Synthesis of zeolite A using kaolin from Ethiopia and its application in detergents. New J. Chem..

[29] The World Bank. The World Bank In Ethiopia. 1–3 (2019). Available at: https://www.worldbank.org/en/country/ethiopia/overview. (Accessed: 20th September 2019)

[30] World Economic Forum. Does foreign aid always help the poor? 1–5 (2015). Available at: https://www.weforum.org/agenda/2015/10/does-foreign-aid-always-help-the-poor/. (Accessed: 19th September 2019)

[31] Saint W (2004). Higher education in Ethiopia: The vision and its challenges. J. High. Educ. Africa.

[32] Tessema KA (2009). The unfolding trends and consequences of expanding higher education in Ethiopia: Massive universities, massive challenges. High. Educ. Q..

[33] Tamrat, W. & Teferra, D. Private higher education in Ethiopia: risks, stakes and stocks. *Stud. High. Educ*. 1–15 (2019).

[34] AKYÜZ M, ROBERTS DJ (2002). Determination of linear alkylbenzene sulphonates and their biodegradation intermediates by isocratic RP-HPLC. Turkish J. Chem..

[35] Wangkarn S, Soisungnoen P, Rayanakorn M, Grudpan K (2005). Determination of linear alkylbenzene sulfonates in water samples by liquid chromatography–UV detection and confirmation by liquid chromatography–mass spectrometry. Talanta.

[36] Ou ZQ, Jia LQ, He YW, Sun TH, Shang DS (1996). Extraction and HPLC determination of LAS in water and soil. Chinese J. Ecol..

[37] Benesty Jacob, Chen Jingdong, Huang Yiteng, Cohen Israel (2009). Pearson Correlation Coefficient. Noise Reduction in Speech Processing.

[38] Kearns JP, Wellborn LS, Summers RS, Knappe DRU (2014). 2, 4-D adsorption to biochars: Effect of preparation conditions on equilibrium adsorption capacity and comparison with commercial activated carbon literature data. Water Res..

[39] Lee Y (2013). Characteristics of biochar produced from slow pyrolysis of Geodae-Uksae 1. Bioresour. Technol..

[40] Yimer, J., Yadav, O. P., Kebede, T. & Mohammed, J. Kinetics and Equilibrium Study of Adsorption of Phenol Red on TEFF (Eragrostis teff) Husk Activated Carbon. (2014).

[41] HKSAR. *Annual Reprot of HKSAR*. (2001).

[42] Ghunmi LA (2008). Quantitative and qualitative characteristics of grey water for reuse requirements and treatment alternatives: the case of Jordan. Water Sci. Technol..

[43] World Bank. *‘The World Bank Data Catalog’*. (2019).

[44] Wolde, W. *et al*. Physical And Fiscal Water Loss (In Case Of Institute Of Technology Campus, Ambo University). (Ambo University, 2018).

[45] Bhagat, Tiyasha, Welde, Tesfaye, Tung, Al-Ansari, Salih, Yaseen (2019). Evaluating Physical and Fiscal Water Leakage in Water Distribution System. Water.

[46] Katsoyiannis IA, Zouboulis AI (2004). Application of biological processes for the removal of arsenic from groundwaters. Water Res..

[47] Gimbel, R., Graham, N. & Collins, M. R. *Recent progress in slow sand and alternative biofiltration processes*. (IWA Publishing, 2006).

[48] McGhee, T. J. & Steel, E. W. *Water supply and sewerage*. 6, (McGraw-Hill New York, 1991).

[49] Human & Environmental Risk Assessment (HERA). *Alcohol Ethoxylates*. (2009).

[50] CAWST. Biosand Filter Construction Manual. *Cent. Afford. Water Sanit. Technol*. 2 (2012).

[51] Nascimento, C. O. C., Veit, M. T., Palácio, S. M., Gonçalves, G. C. & Fagundes-Klen, M. R. Combined Application of Coagulation/Flocculation/Sedimentation and Membrane Separation for the Treatment of Laundry Wastewater. *Int. J. Chem. Eng*. **2019**, (2019).

[52] Babaei F (2019). Removal of linear alkylbenzene sulfonate and turbidity from greywater by a hybrid multi-layer slow sand filter microfiltration ultrafiltration system. J. Clean. Prod..

[53] Faria CV, de Delforno TP, Okada DY, Varesche MBA (2019). Evaluation of anionic surfactant removal by anaerobic degradation of commercial laundry wastewater and domestic sewage. Environ. Technol..

[54] Ji, G. *et al*. Combined UMBAF−MBAF process treating detergent wastewater. *Water Environ. Res*. (2019). 10.1002/wer.1091 30793418

[55] Watiniasih, N. L., Purnama, I. G. H., Padmanabha, G., Merdana, I. M. & Antara, I. N. G. Managing laundry wastewater. In *IOP Conference Series: Earth and Environmental Science* **248**, 12084 (IOP Publishing, 2019).

[56] Al-Gheethi, A. A. *et al*. Efficiency of moringa oleifera seeds for treatment of laundry wastewater. In *MATEC Web of Conferences* **103**, 6001 (EDP Sciences, 2017).

[57] DWAF, D. of water and forestry. *Water Quality Guidelines Agricultural Use: Irrigation Volume 4*. **4**, (1996).

[58] George, P. R. Agricultural Water Quality Criteria Irrigation Aspects. *Dep. Agric. Food, West. Aust*. 16 (1983).

[59] Kannel PR, Lee S, Lee Y-S, Kanel SR, Pelletier GJ (2007). Application of automated QUAL2Kw for water quality modeling and management in the Bagmati River, Nepal. Ecol. Modell..

[60] Esayas, B., Simane, B., Teferi, E., Ongoma, V. & Tefera, N. Climate Variability and Farmers’ Perception in Southern Ethiopia. *Adv. Meteorol*. **2019**, (2019).

[61] Al-Hamaiedeh H, Bino M (2010). Effect of treated grey water reuse in irrigation on soil and plants. Desalination.

[62] Ghaitidak DM, Yadav KD (2013). Characteristics and treatment of greywater—a review. Environ. Sci. Pollut. Res..

[63] Luo J (2019). Promoting the anaerobic production of short-chain fatty acids from food wastes driven by the reuse of linear alkylbenzene sulphonates-enriched laundry wastewater. Bioresour. Technol..

[64] Motteran F, Braga JK, Silva EL, Varesche MBA (2016). Kinetics of methane production and biodegradation of linear alkylbenzene sulfonate from laundry wastewater. J. Environ. Sci. Heal. Part A.

